# Supplementary material for: Patterns of Intron Gain and Loss in Fungi
Source: PLoS Biol. 2004 Nov 30;2(12):e422. doi: 10.1371/journal.pbio.0020422 (PMC532390; doi:10.1371/journal.pbio.0020422)
Supplement: Table S1 — Also available at http://genes.mit.edu/NielsenEtAl/. (4.3 MB ZIP). [file pbio.0020422.st001.zip › NielsenEtAl/html/1282.html]

AN5057.1.NCU03246.1.MG04637.1.FG00543.1


```
 CLUSTAL W (1.82) Multiple Sequence Alignments - Introns Inserted


Sequence 1: NCU03246.1	631 aa
Sequence 2: MG04637.1	631 aa
Sequence 3: FG00543.1	633 aa
Sequence 4: AN5057.1	595 aa
Alignment Length: 676 aa
Number Identitical Residues: 262 aa
Alignment Score (without introns) 13544


MG04637.1 	----------------MGQVIEYIEN1RLYLASYVNPPEADTLFPYPE-PPTRSPTKRSR
NCU03246.1	MAPSSRH------SSNLGQIIEYIPD1RLYLASYSEPPTEHTLFPYPD-PEPRSPRKRSQ
FG00543.1 	MAPSSRHPQLQPQPQSMAQIVEYIPD1RLYLAAYNVAPTADTPFPYPEEPAPVSPRKRTT
AN5057.1  	MAGSTAG---------YGQIIEYIQD1KLYLASYDHTPDAKTPFPYPA-EQPKSPSKRRA
          	 : ::            .*::*** : :****:*  .*  .* ****    . ** **  

MG04637.1 	---AAVEPTPVAARKQPYYFSIDDSLLYNAFHHDFGPLHIGHLYRFALEFHDILGAKENK
NCU03246.1	---RTID--QVTERKKPYYFTVDDTLLYNAFHHDFGPFHIGHLYRFALQFHEILGAKENK
FG00543.1 	RLAAGATPLAKPSRPQPCYFTVDDTLLYNAFHHDFGPLHIGHLYRFAIQFHDILGAKQNK
AN5057.1  	-------QASPSKKRSPVYFTVDDTLLYNSFHADFGPLHIGHLYRFAVHFHDLLAA--NN
          	           . : .* **::**:****:** ****:*********:.**::*.*  *:

MG04637.1 	DRPVVFWSRADPRS1RANAACVLACYMVLIQSWPPHLA~LAPIAQVDPPLMPFRDAGYST
NCU03246.1	DRPVVFWSRADPR-~----------------------T1LAPVAQVDPPLMPFRDAGYSQ
FG00543.1 	ERPIVFYSAADPKS1RANAACMLACYMVLIQNWPPHLA~LAPIAQVDPPLMPFRDAGYSQ
AN5057.1  	DRAIVFYSRTDARS~RANAACLVACYMVLIQSWPPHLA~LAPIAQADPPYMPFRDAGYSQ
          	:*.:**:* :*.::  :.::.  :.     .. ..  : ***:**.*** ********* 

MG04637.1 	ADYGITVQDVVYGVWKAKEEGCVMLDSFDLGE~YEKYERVENGDFNWITPHFLAFASPMH
NCU03246.1	ADYGITVQDVVYGVWKAKEEGCCVLETFDLDE~YEKFERVEHGDFNWITPNFLAFASPQH
FG00543.1 	ADYGISVQDVVYGVWKAKEEKCCDLDNFDLDE~YERFERVEHGDFNWITPHFLAFASPQH
AN5057.1  	ADFILNIQDVVYGVWKAKEQGVCGLRDFSLEE2YEKFERVDMGDFNWISPHFLAFASPQH
          	**: :.:************:    *  *.* * **::***: ******:*:******* *

MG04637.1 	YPVDRVTETMGEAYKLLPKTIADVNKHPDLPDPFKNVLCHFSEKNIGLVVRLNSQLYSPS
NCU03246.1	TPVAPTPEGT-EQWSLLPKTLAAVDAHPTLPQPFKNVLRHFSERDIGLVVRLNSQLYNPS
FG00543.1 	APVQKVTEGS-ELFPLLPRTIAAVDAHPKLPKPFKNVLKHFSEKNIGLVVRLNSQLYSPS
AN5057.1  	QPVAPIPRDS-PEYAALPSTVSEVRSS-RLPLPFKNVLEHFATRGVGLVVRLNSELYSPS
          	 **   ..     :  ** *:: *     ** ****** **: :.:********:**.**

MG04637.1 	YFESLGIRHLDMIFEDGTCPPLSLVRKFIRLAHETITIRKRGIAVHCKAGLGRTGCLIGA
NCU03246.1	YFEALGINHIDMIFEDGTCPTLSTVRKFIRMAHETIS-RKKGIAVHCKAGLGRTGCLIGA
FG00543.1 	YFEALGIQHLDMIFDDGTCPSLTTVRKFIRLAHETITVRKKGIAVHCKAGLGRTGCLIGA
AN5057.1  	YFTALGISHIDMIFEDGTCPPLPLVKKFIRMAHEMINVKHKAIAVHCKAGLGRTGCLIGA
          	** :*** *:****:*****.*. *:****:*** *. :::.******************

MG04637.1 	YLIYRHGFTADEVISYMRFMRPGMVVGPQQHWLHLNQGVFREWWVEERVARKLRKELAAA
NCU03246.1	YLIYRHGFTANEVIAYMRFMRPGMVVGPQQHWLHLNQGIFREWWIEERVERRLRKEMAAA
FG00543.1 	YLIYRHGFTANEVISFMRFMRPGMVVGPQQHWLHLNQGTFREWWVEERIERRLRREMAAA
AN5057.1  	YLIYRYGFTANEVIAFMRFMRPGMVVGPQQHWLHLNQGSFREWWFEDCMKEKLAQMQP--
          	*****:****:***::********************** *****.*: : .:* :  .  

MG04637.1 	AQNTANTG---VPSTPIRAMQKASLGRTSATQASTPPSKS---RTPLGEMDTES---NTV
NCU03246.1	AAAAAQANGTAHPSTPVRAVSKTSLGRSSSKNGVSTPSNRGSVRTPLGEIDRESARDNHI
FG00543.1 	NP---------IPSTPIRAMQKTTLRNG---QASTPPNRSPSNRTPLSEVDHDR---NNI
AN5057.1  	-----------NPVTPGRSPAKHRAAAV-----TTPPQNGHSKRSALGEIDNNE----AT
          	            * ** *:  *            :.*..  : *:.*.*:* :       

MG04637.1 	GVQEDYLPAPTPGQPRKTGRNPSGRHHPYSRNDAGTKGEEQ-----QHTSESISVHRSTN
NCU03246.1	GVQEDYLPAPTPGQPRKTQRGAVDRQKGYGRGPIQTTVEEEPVPMTEVTTEVITVHKSSR
FG00543.1 	GVQEDYLPAPTPGQPRKTAR---DRHHPYQRSISGGVPPEAQYPVDQEADLIAKYTQASG
AN5057.1  	PIYDDNLPAPTPGQPRKSHRKD-SRHHPYSRTASGSLVVDK---------DTRKTRRSTD
          	 : :* ***********: *   .*:: * *        :             .  ::: 

MG04637.1 	--ESDEEWHMRMAR---TSTRASPNEKKQRSASHTTTTTTTHHVYSSSSTYVDNDASNDI
NCU03246.1	GPSSEEEWNLRRSRRRQSAAPTSPGQPPERSVTHQTATTKTT-VY----QVMDNDASNDI
FG00543.1 	--ESDEELHLRMRR------RKATSQSPARSE-KTRSVSQTTAIY-----TIDNDASHDA
AN5057.1  	SSESEEETQLRMLAK-QRSSKSPAASPGQRSISYSATVTASY--------TLNDDIHEDR
          	...*:** ::*     . ::  .. .   ** :   :.: :          :::*  .* 

MG04637.1 	ENIGSSVGRTKSVKEEAR-SSASGVLAKVRG---VERS--GSTRRAAASTTVGKEPTGIR
NCU03246.1	ENIGTAAARIKAHDHAPRTSSASGVLTKIRGNTSAKRHPPGSVREAGVRKTSGRVGS-IG
FG00543.1 	ENIGSVRS-----KYVERVPSTPGVLAKVRG-SKRQGESPLRAKESGVRKTSGRVGS---
AN5057.1  	ENWGGAAQPPK--TPVTSKTSGAVSVSKVRS---SSRRVTGESK--GVRKPSGRIGS---
          	** *      .        .* .  ::*:*.    .   .   :  .. .. *:  :   

MG04637.1 	KTSGRVASAHAGAVRKASGL
NCU03246.1	NST--TSPAAVSAMRKISGM
FG00543.1 	-ASHSTSVSAASTARKVSGA
AN5057.1  	---------TGSPVRVKAQA
          	           .. *  :
```
